# Technology versus tradition: a non-inferiority trial comparing video to face-to-face consultations with a physiotherapist for people with knee osteoarthritis. Protocol for the PEAK randomised controlled trial

**DOI:** 10.1186/s12891-020-03523-8

**Published:** 2020-08-07

**Authors:** Rana S. Hinman, Alexander J. Kimp, Penny K. Campbell, Trevor Russell, Nadine E. Foster, Jessica Kasza, Anthony Harris, Kim L. Bennell

**Affiliations:** 1 Centre for Health, Exercise and Sports Medicine, Department of Physiotherapy, School of Health Sciences, Faculty of Medicine Dentistry & Health Sciences, The University of Melbourne, Melbourne, Australia; 2 RECOVER Injury Research Centre, The University of Queensland, Brisbane, Australia; 3 Primary Care Centre Versus Arthritis, School of Primary, Community and Social Care, Keele University, Staffordshire, UK; 4 School of Public Health and Preventive Medicine, Monash University, Melbourne, Australia; 5 Centre for Health Economics, Monash Business School, Monash University, Melbourne, Australia

**Keywords:** Osteoarthritis, OA, Knee, Telehealth, Telerehabilitation, Rehabilitation, Physiotherapy, Clinical trial, RCT, Musculoskeletal, Pain, Exercise, Physical activity, Non-inferiority

## Abstract

**Background:**

Knee osteoarthritis (OA) is a global problem that causes significant pain and physical dysfunction, substantially impacting on quality of life and imposing enormous cost to the healthcare system. Exercise is pivotal to OA management, yet uptake by people with knee OA is inadequate. Limited access to appropriately skilled health professionals, such as physiotherapists, for prescription of an exercise program and support with exercise is a major barrier to optimal care. Internet-enabled video consultations permit widespread reach. However, services offering video consultations with physiotherapists for musculoskeletal conditions are scant in Australia where there is typically no Government or private health insurer funding for such services. The paucity of robust evidence demonstrating video consultations with physiotherapists are clinically effective, safe and cost-effective for knee OA is hampering implementation of, and willingness of healthcare policymakers to pay for, these services.

**Methods:**

This is an assessor- and participant-blinded, two-arm, pragmatic, comparative effectiveness non-inferiority randomised controlled trial (RCT) conducted in Australia. We are recruiting 394 people from the community with chronic knee pain consistent with a clinical diagnosis of knee OA. Participants are randomly allocated to receive physiotherapy care via i) video-conferencing or; ii) face-to-face consultations. Participants are provided five consultations (30–45 min each) with a physiotherapist over 3 months for prescription of a home-based strengthening exercise program (to be conducted independently at home) and physical activity plan, as well as OA education. Participants in both groups are provided with educational booklets and simple exercise equipment via post. The co-primary outcomes are change in self-reported i) knee pain on walking; and ii) physical function, with a primary end-point of 3 months and a secondary end-point of 9 months. Secondary outcomes include changes in other clinical outcomes (health-related quality of life; therapeutic relationship; global ratings of change; satisfaction with care; self-efficacy; physical activity levels), time and financial costs of attending consultations, healthcare usage and convenience. Non-inferiority will be assessed using the per-protocol dataset.

**Discussion:**

Findings will determine if video consultations with physiotherapists are non-inferior to traditional face-to-face consultations for management of people with knee OA.

**Trial registration:**

Australian New Zealand Clinical Trials Registry, ACTRN12619001240134. http://www.anzctr.org.au/Trial/Registration/TrialReview.aspx?id=377672&isReview=true

## Background

Arthritis and musculoskeletal conditions are an Australian National Health Priority Area, and more prevalent in Australia than any other, including cancer, diabetes and obesity [1]. Osteoarthritis, particularly of the knee, is a leading cause of pain and disability in Australia, and the 12th highest contributor to global disability [2]. Around 2.1 million Australians (1 in 11 people) have OA, with a 58% increase expected by 2032 due to population ageing and rising obesity rates [1]. This reflects international data showing that OA accounted for 303.1 million prevalent cases across the globe in 2017 [3], with a 9.3% increase in age-standardised point prevalence since 2010. Knee OA can be extremely debilitating. Pain is a major symptom and it may become persistent and more limiting over time. Physical function can become increasingly impaired and may impact substantially on quality of life and ability to participate in social, leisure and occupational activities. Health expenditure on OA in Australia in 2012 was $3.75 billion, with most costs related to conservative and surgical treatments, lost productivity and loss of quality of life. With the aging and increasing obesity of the world’s population, a large increase in demand for health services for knee OA is expected in the future.

There is no cure for OA. Treatments to reduce symptoms and delay or prevent joint replacement are critical. Clinical guidelines for management of knee OA emphasise non-drug non-surgical strategies [4–7] that focus on self-help and patient-driven options rather than clinician-delivered passive therapies. In particular, advice and information for self-management, exercise and weight control (if required) are core management, with drugs, injections and manual therapy considered adjunctive to core treatments [4–7]. Exercise is advised for all people with knee OA irrespective of age, OA severity, pain, function and comorbidities [6]. The benefits of exercise for knee OA on pain, physical function and quality of life are well-established [8, 9]. Given that muscle weakness is widespread in knee OA [10], muscle strengthening is an important component of exercise programs for people with the condition.

Knee OA is mostly managed in primary care settings, with physiotherapists frequently involved in provision of care. Physiotherapy is traditionally provided during face-to-face consultations in the clinic. However, for many people, access to physiotherapy is hampered by geographical isolation and/or limited physiotherapy services. This is particularly a problem in regional and remote areas where services are often limited or non-existent. Face-to-face consultations also pose an infection risk for both the physiotherapist and the patient. This has been highlighted by the COVID-19 pandemic where many physiotherapy practices across the globe have restricted or ceased face-to-face consultations and instead shifted towards telehealth, in order to safeguard the health of both patients and staff.

We conducted the first RCT evaluating efficacy of video consultations with a physiotherapist for exercise management of knee OA [11]. We recruited 148 older people around Australia and compared video consultations (delivered by 8 different physiotherapists) combined with a self-directed online pain coping skills program, versus online education. Findings showed that the intervention significantly improved knee pain and physical function by clinically meaningful amounts at 3 months, and that benefits were sustained at 9 months. Furthermore, nested qualitative research showed that video consultations were acceptable to both patients and physiotherapists [12]. Patient convenience, flexibility, empowerment to self-manage and positive therapeutic relationships were emphasised by both patients and physiotherapists. Patients were very satisfied with the care they received and believed it was effective for them personally. These findings reflect survey data in people with knee OA [13] and in patients attending hospital-based physiotherapy screening clinics [14], as well as RCT findings in patients following joint replacement [15]. Collectively, research indicates most patients are willing to engage in video consultations for chronic musculoskeletal conditions. However, there is no research comparing the effectiveness of video-conferencing with physiotherapists to face-to-face consultations for people with knee OA.

The primary aim of this RCT is to determine if video-conferencing consultations are non-inferior to face-to-face consultations with a physiotherapist for improving knee pain on walking and/or physical function at 3 months (primary time-point) and 9 months (secondary time-point), in people with knee OA. We hypothesise that video consultations are not inferior for improving knee pain on walking and/or physical function compared to face-to-face care in people with knee OA. Secondary aims are to compare the clinical effectiveness (health-related quality of life, therapeutic relationship, global ratings of change, satisfaction with care, self-efficacy, physical activity levels), cost-effectiveness (participant-level travel time and travel-related costs, health care usage) and convenience of video-conferencing consultations to face-to-face consultations, as well as explore potential moderators of treatment effect.

## Methods

### Study design

A multi-site, two-arm, parallel, pragmatic, comparative effectiveness non-inferiority RCT is being conducted, with a health economic analysis. This protocol has been developed according to the SPIRIT statement [16]. The RCT was prospectively registered with the Australian and New Zealand Clinical Trials Registry (ACTRN12619001240134). Findings of the trial will be reported according to CONSORT guidelines for reporting of non-inferiority trials [17] and non-pharmacological interventions [18], and the Australia & New Zealand Musculoskeletal (ANZMUSC) Clinical Trial Network governance and publication policies (https://anzmusc.org).

### Participants

A total of 394 people with chronic knee pain, consistent with a clinical diagnosis of knee OA, will be recruited. Participants are recruited from the community (in geographical locations surrounding our trial physiotherapists) in Victoria, Queensland, and New South Wales via community advertisements, print/radio/social media, clinicians and our volunteer database. Volunteers are initially screened by an online form, then over the phone by the Trial Coordinator. Volunteers uncomfortable with online screening can instead call a telephone number and proceed directly to phone screening. Participants are eligible for the study if they meet the following inclusion criteria:

i. meet National Institute for Health and Care Excellence [6] clinical criteria for OA;
i. age ≥ 45 years;
ii. report activity-related knee joint pain and;
iii. report no morning stiffness or morning knee stiffness lasting ≤30 mins;
ii. report history of knee pain ≥3mths;
iii. report knee pain on most days of the past month;
iv. report an average pain score ≥ 4 on an 11-point numeric rating scale during walking over the previous week;
v. report difficulty walking and climbing stairs;
vi. access to a device with internet connection;
vii. willing and able to travel to the nearest trial physiotherapist if required; and
viii. pass the Exercise and Sports Science Australia stage 1 pre-exercise screening questions [19].

Participants are excluded if they:

i. are unable to speak English;
ii. are on a waiting list/planning for knee/hip surgery in next 12 months;
iii. have had previous arthroplasty on affected knee;
iv. report recent knee surgery (past 6 months);
v. are currently consulting/ed. a physiotherapist or doing strengthening exercise for their knee (past 6 months);
vi. report any inflammatory arthritis (e.g. rheumatoid arthritis);
vii. report any neurological condition affecting lower limbs; and/or
viii. report any unstable/uncontrolled cardiovascular condition.

Anyone who i) reports a fall (past 12 months) or is house-bound due to immobility; or ii) who fails the Exercise and Sports Science Australia stage 1 pre-exercise screening questions is asked to obtain clearance from a general practitioner to participate. In cases where an eligible participant has bilateral knee symptoms, the most symptomatic knee is considered the study knee.

### Procedures overview

**Fig. 1:**
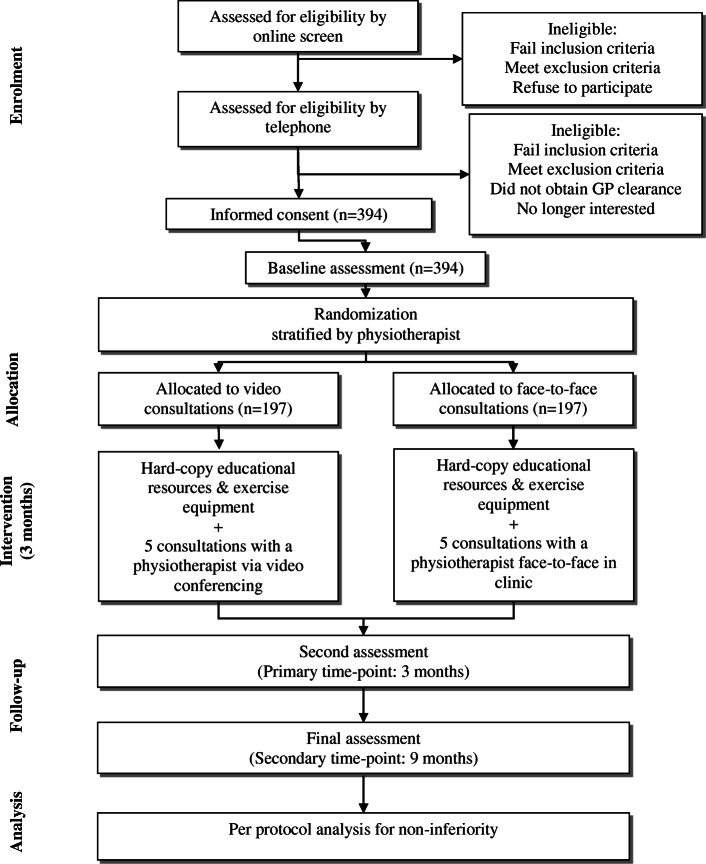
Flow of participants through the RCT

The flow of participants through the RCT is outlined in Fig. 1. All potential participants receive oral and written information about the purposes, potential risks and processes involved in the study from the Trial Coordinator. Once participants pass the telephone screening process, which involves a detailed verbal description of the project, they are sent a Plain Language Statement and Consent Form in the post or by email. Ethical approval has been obtained from the University of Melbourne Human Research Ethics Committee (HREC No. 1953585.1). All participants provide written informed consent.

All outcomes are participant-reported. Participants complete outcome assessments via electronic survey (REDCap) or paper-based (returned via mail for the minority that may request this). In addition, all participants record attendance at, and travel information about, physiotherapy consultations using a log book which is mailed back to research staff. As a reminder, research staff contact any participant who has failed to submit a survey and/or return a log book by the due date. Every effort is being made to minimize loss of data, including collection of primary outcome data over the telephone if necessary.

Immediately upon receipt of the signed consent form and completed baseline survey, a participant is randomized to either video-conferencing or face-to-face consultations with the trial physiotherapist of their choice (usually selected on the basis of geographical location). Participants are provided five physiotherapy consultations over a 12-week intervention period. Research staff book the first two consultations on behalf of the participant with the physiotherapist’s clinic at mutually agreeable times. Thereafter, the physiotherapist and participants arrange subsequent bookings together at the conclusion of each consultation.

### Randomisation, blinding and allocation concealment

The randomisation schedule was prepared by a biostatistician using permuted block sizes 6 to 12 and a randomisation ratio of 1:1, stratified by trial physiotherapist. Where physiotherapists practice at two different clinic locations, randomisation is stratified by location within the physiotherapist. The randomisation schedule is stored on a password-protected website (REDCap) at the University of Melbourne and maintained by a researcher not involved in either participant recruitment or administration of primary/secondary outcome measures. Group allocation is revealed by this same researcher after baseline assessment has been completed.

Participants are blinded to group allocation by a process of limited disclosure. Participants are informed that physiotherapy services in the ‘real-world’ may be provided in a variety of settings and models of delivery, such as face-to-face care, telephone consultations, video-conferencing, individual consultations, group classes, community health services, rehabilitation services, private practices and hospital settings and home visits. They are informed that the purpose of the RCT is to compare the effectiveness of two different models of physiotherapy service. Although participants are informed they will receive a series of consultations with a physiotherapist (for education, strengthening exercise and a physical activity plan), they are not told that video-conferencing is being compared to face-face individual consultations. Participants will not be informed about the study hypotheses, or the model of care received by the comparator group, until the study is completed, at which time they will also be provided a lay summary of findings. As all outcomes are participant-reported, and participants are blinded, this study is thus also considered assessor-blinded. It is not possible to blind physiotherapists as they are providing consultations to both trial arms (in order to ensure physiotherapist-related factors such as personality, clinical practice experience etc. are similar across groups and cannot confound results). Statistical analyses will be performed in a blinded manner.

### Physiotherapy care

#### Physiotherapists

Fifteen practicing musculoskeletal physiotherapists in private practice in Victoria and Queensland were recruited via Australian Physiotherapy Association electronic communications channels, and our own clinical networks. These physiotherapists deliver care to participants randomized to both trial arms. Eligibility criteria for the physiotherapists to participate were:

- Current registration to practice as a physiotherapist;
- Have a receptionist at their clinical practice to facilitate patient bookings and communication with research staff;
- Have access to a desktop or laptop computer with internet connection and suitable work-space for private video consultations in their clinical rooms;
- Have an Australian Business Number;
- Have some previous experience and confidence using video conferencing software (e.g. Skype, Zoom, FaceTime);
- Willing to undertake trial training requirements; and
- Be willing and available to participate in the RCT until end of 2021.

#### Physiotherapist training

Mandatory training was undertaken by all physiotherapists prior to being allocated a trial participant. Training included:

- Bespoke self-directed e-learning modules (delivered on the University of Melbourne Learning Management System) about best-practice OA management, telehealth (including delivery of care via Zoom video-conferencing) and trial procedures, including the structured physiotherapy treatment protocol. Physiotherapists were told it would take approximately 5 h to work through all e-learning modules, which they were encouraged to complete at their own pace, ideally over 4 weeks. The PEAK Training Program e-learning modules have since been adapted, and released, for widespread use by clinicians outside of the trial and access is available to users from all over the globe via https://healthsciences.unimelb.edu.au/departments/physiotherapy/about-us/chesm/news-and-events/peak-training-program/);
- A mock initial consultation via video-conferencing with a researcher acting as a patient, who provided immediate feedback on performance;
- Four video consultations, with two pilot patients with knee pain recruited by research staff (an initial and a follow-up call for each patient), to practice video consultation skills. Physiotherapists completed a self-reflection exercise upon completion of the pilot consultations (What went well? What didn’t go so well? What would I do differently in future?). Research staff conducted “spot” checks of consultation recordings and provided feedback to individual physiotherapists regarding performance after all pilot consultations were complete;
- A video-conference with research staff to answer any questions about trial procedures.

A hard copy of the trial protocol and procedures relevant to the physiotherapist (incorporated into a single physiotherapist trial manual), as well as a copy of each participant resource (see below) was also provided to each physiotherapist.

#### Participant resources

Each trial participant is mailed a “welcome pack” of resources to facilitate the management plans that physiotherapists enact during the consultations. These resources include:

- Four information booklets:
- “Preparing for your consultations” (information about consultations, instructions on how to use Zoom video-conferencing and activity tracker);
- “Osteoarthritis Information” (information about knee OA, common management options, exercise and physical activity, weight loss, pain management, sleep and success stories);
- “Exercise Booklet” (strengthening exercise instructions and photos, including information on progressing exercises, managing flare-ups and dealing with set-backs); and
- “Knee Plan and Log Book” (templates to record details of management plans agreed upon at each consultation and monitor progress with exercise and physical activity goals).
- Four coloured elastic resistance bands (red, green, blue, and black) for home-based strengthening exercises; and
- A wearable activity tracker to facilitate physical activity plans (Mi Band 4, Anhui Huami Information Technology Co. Ltd., China) or pedometer (Omron Healthcare, USA).

#### Consultations

In both trial arms, five consultations are offered with the physiotherapist over 3 months (at approximately weeks 1, 2, 4, 7, 10), each lasting 30 min except the first which is 45 min. Participants are asked to complete an electronic pre-consultation survey prior to their first physiotherapy consultation, which ascertains information about their knee symptoms, previous treatments, activity levels and personal goals. This survey is forwarded to the physiotherapist prior to the first consultation.

Table 1 provides an overview of the main components of each physiotherapy consultation. Consultations have been informed by the Behaviour Change Wheel [20] (including behaviour change techniques relevant for exercise and physical activity [21, 22], Table 2) and based on the research team’s prior research into exercise and physical activity for knee OA [11, 23–27]. In summary, physiotherapists aim to prescribe an individualised exercise program comprising 5–6 strengthening exercises to be performed at home three times/week, including two quadriceps exercises, one hip/gluteal exercise, one hamstrings/gluteal exercise, one calf exercise, and one other as appropriate. Exercises are selected from an Exercise Booklet containing 37 exercises in total (Table 3), many with a range of potential variations to make them harder or easier as required. The intensity for the strengthening exercises is aimed at 5–7 out of ten (or, “hard to very hard”) on the modified Borg Rating of Perceived Exertion (RPE) scale [28] for strength training. Review and modification of the strengthening program occurs at each consultation. Progression is guided by American College of Sports Medicine principles [29] via adjustments to repetitions, direction, and speed of movements; increasing resistance; and/or changing stance surface.

**Table 1:** Outline of the main components of each of the five consultations with the physiotherapist

	Initial consultation (45 mins)- WEEK 1	Consultation 2 (30 mins)- WEEK 2	Consultation 3 (30 mins)- WEEK 4	Consultation 4 (30 mins)- WEEK 7	Consultation 5 (30 mins)- WEEK 10
**Assessment**	15 mins	5 mins	5 mins	5 mins	5 mins
	Introduction and setting expectations	Checking in … .	Checking in … .	Checking in … .	Checking in … .
	Review pre-consultation survey- choose questions for future reassessment.	- changes in knee pain	- changes in knee pain	- changes in knee pain	- changes in knee pain
		- how they have managed with strengthening exercises	- how they have managed with overall program	- how they have managed with overall program	- how they have managed with overall program
	Subjective information as relevant.	- adverse events?	- adverse events?	- adverse events?	- adverse events?
	Functional observation: walking, squatting, sit to stand, single leg standing balance, anything else as relevant.	- comments/questions arising from discussions last time.	- comments/questions arising from discussions last time.	- comments/questions arising from discussions last time.	- comments/question s arising from discussions last time.
			Reassess questions from pre-consultation survey.		Reassess questions from pre-consultation survey.
			Re-assess sit to stand and any other functional tasks as required.		
					Re-assess sit to stand and any other functional tasks as required.
					Check progress with goals.
**Education**	10 mins				
	Understanding OA.				
	Benefits of exercise/physical activity.				
**Strengthening exercises**	15 mins	10 mins	10–15 min	10–15 min	15 mins
	Choose a program of 3 exercises from booklet (1 quadriceps; 1 hip/gluteal; 1 hamstrings/gluteal)	Review progress.	Review progress.	Review progress.	Review progress.
		Check adherence in Log Book.	Check adherence in Log Book.	Check adherence in Log Book.	Check adherence in Log Book.
		- Congratulate adherence.	- Congratulate adherence.	- Congratulate adherence.	- Congratulate adherence.
	Prescribe variation (if necessary) and dosage.	- Discuss reasons for non-adherence & troubleshoot.	- Discuss reasons for non-adherence & troubleshoot.	- Discuss reasons for non-adherence & troubleshoot.	- Discuss reasons for non-adherence & troubleshoot.
	Watch patient perform one set of each exercise & ensure they are working at hard to very hard level.				
		Review current exercises & modify/progress as required & add 2–3 more to program (max 6 in total; 2 quadriceps; 1 hip/gluteal; 1 hamstrings/gluteal; 1 calf; 1 optional extra).	Review current exercises & modify/progress as required.	Review current exercises & modify/progress as required.	Review current exercises & modify/progress as required.
	Discuss exercising with pain/flare-ups.		Watch patient perform one set of any new exercises & ensure they are working hard to very hard.	Watch patient perform one set of any new exercises & ensure they are working hard to very hard.	Watch patient perform one set of any new exercises & ensure they are working hard to very hard.
	Instruct use of Log Book.				
					Check patient knows how to change/progress their program over the next 6 months.
		Watch patient perform one set of each new exercise & ensure they are working hard to very hard).			
					Advise patient to continue exercise program for next 6 months.
**Education**		5 mins			
		Physical activity. Activity pacing.			
**Physical activity**	5 mins	10 mins	5–10 min	5–10 min	5 mins
	Check patient has activity tracker set up and can use it.	Review daily step count recorded in Log Book.	Review daily step count recorded in Log Book.	Review daily step count recorded in Log Book.	Review daily step count recorded in Log Book.
	Instruct patient to wear activity tracker every day for next week and record daily steps as a baseline for developing a daily step goal at the next visit.	Set daily step goal (may be maintain or increase from baseline).	Review progress with physical activity plan.	Review progress with physical activity plan.	Review progress with physical activity plan.
					- Congratulate adherence.
		Agree on physical activity plan to achieve steps and/or increase/maintain intensity of activity.	- Congratulate adherence.	- Congratulate adherence.	- Discuss reasons for non-adherence & troubleshoot.
			- Discuss reasons for non-adherence & troubleshoot.	- Discuss reasons for non-adherence & troubleshoot.	
					Set an ongoing physical activity plan that patient can manage in daily life (may/may not include step goals & use of activity tracker based on patient choice).
	Instruct use of Log Book.	Ask patient to identify potential barriers & plan strategies for overcoming them.	Set daily step goal for coming weeks (may be to maintain or increase from previous weeks). Agree on physical activity plan to achieve step goal and/or increase/maintain intensity of activity.	Set daily step goal for coming weeks (may be to maintain or increase from previous weeks). Agree on physical activity plan to achieve step goal and/or increase/maintain intensity of activity.	
					Check patient knows how to change/progress their program as required.
					Advise patient to continue physical activity for next 6 months.
**Education**			5 mins	5 mins	5 mins
			Understanding & managing your pain.	Weight loss for OA (relevant for all patients, even those of healthy body weight)	Dealing with lapses & set-backs.
					Encourage ongoing use of Log Book and activity tracker where possible.
**Participant ‘homework’**	Pre-reading:	Pre-reading:	Pre-reading:	Pre-reading:	Encourage reading of:
	Physical activity.	Barriers to exercise and physical activity.	Weight loss for OA- if appropriate for the individual.	Modifying your exercise program.	Success stories.
	Activity pacing.	Understanding & managing pain.		Dealing with lapses & set-backs.

**Table 2:** Main behaviour change techniques that are incorporated into the intervention components for both trial arms

**Behaviours:**			
- **Undertake strengthening exercise program**			
- **Undertake negotiated physical activity plan**			
**Behaviour change technique**	**Written information**	**Physiotherapist discussion**	**Other**
**Consequences of behaviour**			
Explanation of benefits of exercise & physical activity.	✓ OA Info booklet	✓ Consult #1	
Explanation that exercise & physical activity will not make joint structural damage worse.	✓ OA Info booklet	✓ Consult #1	
**Goal setting & action planning**			
Use of a plan stating how often to exercise & which exercises to do (including dosage).	✓ Knee plan & log book	✓ Consults #1–5	
Development of specific goals related to patient’s knee problems.		✓ Consult #1 & 5	✓ Pre-consultation survey
Development of specific physical activity & step goals.	✓ Knee plan & log book	✓ Consults #2–5	
**Barrier identification/planning**			
Information & discussion about barriers to exercise & physical activity adherence, including problem-solving.	✓ OA Info booklet	✓ Consults #1–5	
	✓ Knee plan & log book		
	✓ Exercise booklet		
**Behavioural grading & instruction**			
Strengthening exercises are graded in number, intensity and/or difficulty to get progressively harder over time.	✓ Knee plan & log book	✓ Consults #1–5	✓ Four graded resistance bands
	✓ Exercise booklet		
Physical activity is graded in duration, intensity and/or frequency to get progressively harder over time.	✓ OA Info booklet	✓ Consults #1–5	
	✓ Knee plan & log book		
Instruction in where, when and how to perform physical activity	✓ Knee plan & log book	✓ Consults #2–5	
Instruction in where, when and how to perform strengthening exercises	✓ Knee plan & log book	✓ Consults #1–5	✓ Four graded resistance bands
	✓ Exercise booklet		
Demonstration of how to perform strengthening exercises		✓ Consults #1–5	✓ Online video library
Encouragement to join group exercise classes.	✓ OA Info booklet	✓ Consults #2–5	
Encouraged to involve partner or family to join in with exercising & physical activity.	✓ OA Info booklet	✓ Consults #2–5	
**Self-monitoring & feedback**			
Encouraged to self-monitor exercise & physical activity	✓ Knee plan & log book	✓ Consults #1–5	✓ Activity monitor
Physiotherapist review of & and feedback on exercise & physical activity recorded	✓ Knee plan & log book	✓ Consults #2–5	✓ Activity monitor
**Relapse prevention**			
Instruction on how to modify exercise & physical activity during flare-ups	✓ Exercise booklet	✓ Consults #1–5	
Planning for set-backs in physical activity & how to overcome them		✓ Consult #2	
Dealing with lapses & set-backs with exercise & physical activity; use of constructive self-talk	✓ Exercise booklet	✓ Consult #5	
**Pain & emotional control**			
Encouragement to use activity pacing & pain coping activities (eg relaxation, pleasant imagery, mindfulness)	✓ OA Info booklet	✓ Consults #2 & 3	
Tips for healthy sleep	✓ OA Info booklet	✓ Consult #3	
**Prompts**			
Encouraged to use reminders to exercise.	✓ OA Info booklet		
**Rewards**			
Patient encouraged to use self-rewards for achieving exercise & physical activity goals	✓ OA Info booklet	✓ Consults #2–5	
Physiotherapist congratulates adherence to exercise & physical activity		✓ Consults #2–5	
**Social comparison**			
Encourage reading of patient success stories	✓ OA Info booklet	✓ Consult #5	
**Review**			
Review of behavioural goals (exercise & physical activity) at follow-up.		✓ Consults #2–5	
Review of outcomes (pain and function) at follow-up.		✓ Consults #2–5	
Review, supervision and correction of strengthening exercise technique.		✓ Consults #1–5

**Table 3:** Strengthening exercise protocol, with progressions (where applicable)

**Maximum of 6 exercises at any one time, performed three times/week**			
2 quadriceps strengthening exercises			
1 hip abduction/gluteal strengthening exercise			
1 hamstring/gluteal strengthening exercise			
1 calf strengthening exercise			
1 other exercise as appropriate			
**1. Quads strengthening**			
**Knee extension**	Non weight-bearing	Q1. Seated knee extension	**Progression:** Increase resistance with elastic band – red through to black
	Non weight-bearing	Q2. Inner range quads over roll	
**Sit-to-stand**	Weight-bearing	Q3. Sit to stand without using hands	**Progression:** lower chair height, hover above the seat without touching down, add resistance band around knees and push outwards while performing sit to stand
	Weight-bearing	Q4. Asymmetrical chair stands (with more weight on arthritis leg)	
**Steps**	Weight-bearing	Q5. Step-ups	**Progression:** Increase step height
	Weight-bearing	Q6. Forward touch-downs from a step	**Progression:** Increase step height, don’t touch floor
	Weight-bearing	Q7. Step-ups with weight	**Progression:** Increase step height, increase weight
	Weight-bearing	Q8. Forward touch-downs with weight	**Progression:** Increase step height, increase weight
**Wall squats**	Weight-bearing	Q9. Partial wall squats	**Progression:** Halfway hold in bent-knee position, increase the amount of body weight taken through the arthritis knee.
	Weight-bearing	Q10. Split leg wall squats	
**Controlled squats**	Weight-bearing	Q11. Controlled squats (with back of chair support)	
**Controlled knee flexion/extension**	Weight-bearing	Q12. Controlled knee flexion/extension with forwards/backwards sliding of opposite leg	
	Weight-bearing	Q13. Controlled knee flexion/extension with forwards/backwards sliding of opposite leg with elastic band	**Progression:** Increase resistance by changing elastic band colour – red through to black
	Weight-bearing	Q16. Controlled knee flexion/extension with sideways sliding of opposite leg	
	Weight-bearing	Q17. Controlled knee flexion/extension with elastic band and sideways sliding of opposite leg	**Progression:** Increase resistance by changing elastic band colour – red through to black
**Step to single leg balance**	Weight-bearing	Q14. Step to standing balance on semi-flexed knee	
	Weight-bearing	Q15. Step to standing balance on semi-flexed knee with arm movements	
**2. Hip abductor/gluteal strengthening**			
**Standing hip abduction**	Non weight-bearing	HA1. Side leg raises in standing with elastic band.	**Progression**: Increase resistance by changing elastic band colour – red through to black, add halfway hold
	Weight- bearing	HA3. Wall push with opposite leg, standing on straight arthritis leg	**Progression:** Hold weight in hand, increase the hold time
	Weight- bearing	HA4. Wall push with opposite leg, standing on arthritis leg with deeper knee bending	
**Side stepping**	Weight-bearing	HA2. Crab walking with elastic band	**Progression:** Increase resistance by changing elastic band colour – red through to black
**3. Hamstring/gluteal strengthening**			
**Supine bridging**	Weight-bearing	HG1. Bridge with hold	
	Weight-bearing	HG2. Split leg bridge with hold	
	Weight-bearing	HG3. Single-leg bridge with hold	
**Standing knee flexion**	Non weight-bearing	HG4. Hamstring curls standing over bench	
	Non weight-bearing	HG5. Hamstring curls standing over bench with elastic band	**Progression:** Increase resistance by changing elastic band colour – red through to black
**Seated knee flexion**	Non weight-bearing	HG6. Seated knee flexion with elastic band	**Progression:** Increase resistance by changing elastic band colour – red through to black
**Standing hip extension**	Non weight-bearing	HG7. Hip extension with knee bent (90°) standing over a bench	
	Non weight-bearing	HG8. Hip extension with knee straight standing over a bench	
	Non weight-bearing	HG9. Hip extension with knee straight with elastic band standing over a bench	**Progression:** Increase resistance by changing elastic band colour – red through to black
**4. Calf strengthening**			
**Standing plantar-flexion**	Weight-bearing	C1. Double leg calf raises	
	Weight-bearing	C2. Single leg calf raises	
	Weight-bearing	C3. Double leg calf raises over edge of step	
	Weight-bearing	C4. Single leg calf raises over edge of step	
**5. Balance (if appropriate)**			
**Tandem stance**	Weight- bearing	B1. Maintain balance in tandem stance	**Progression:** remove hand support (if required), slowly raise arms in the air, eyes closed
**Natural stance**	Weight- bearing	B2. Maintain balance whilst tapping opposite foot forwards & backwards	
**Single leg stance**	Weight- bearing	B3. Maintain balance in single leg stance	**Progression:** Increase hold time up, slowly raise arms up and down, eyes closed

Physiotherapists also work with the patient to devise an individualised physical activity plan, with the aim of increasing physical activity to, or maintaining it at, recommended levels [30]. Participants use a wearable activity tracker to assist this and the physiotherapist and participant devise individualised step goals that are reviewed and modified (if required) at each consultation. Participants are encouraged to use the “Knee Plan and Log Book” for recording both their strengthening exercise program and physical activity plan after each consultation, and monitoring progress for discussion with the physiotherapist at the next consultation. Education about OA and its management occurs at all sessions, including discussion about the role of exercise and physical activity, including barriers to adherence and strategies for overcoming obstacles. Table 1 outlines the different education topics at each consultation.

Participants are encouraged and advised how to independently progress their exercise program and their physical activity plan between consultations, and beyond, once the physiotherapist consultations have ceased. Participants are encouraged to continue with their strengthening exercises and physical activity plan after the physiotherapist consultations have finished, up until the final outcome measurements are collected at 9 months. Participant use of monitoring and tracking tools (“Knee Plan and Log Book”; wearable activity tracker) is encouraged during the consultations, and beyond, to facilitate ongoing adherence to strengthening exercise and physical activity.

#### Mode of physiotherapy delivery

Face-to-face consultations – participants allocated to this group attend all of their consultations with the physiotherapist in the physiotherapist’s clinical practice rooms. Participants travel to and from the clinic at their own expense. Physiotherapists use their usual clinical methods for teaching the strengthening exercise program (ie demonstration, watching and correcting participant technique). Consultations are audio-recorded using digital audio recorders and uploaded by the physiotherapist to a secure password-protected cloud-based system.

Video consultations - participants allocated to this group attend all of their consultations using the video-conferencing facility of Zoom (Zoom Video Communications, Inc., USA), a commercial cloud-based system accessible from any internet-connected computer, laptop or mobile device. Physiotherapists consult from their clinic using a lap-top or desk-top computer, whilst participants are based at their home (or elsewhere if preferred). Instructions for accessing and downloading Zoom are provided to participants and participants are encouraged to independently set themselves up in readiness for their first consultation. Physiotherapists are given access to a bespoke website containing a video library of all exercises contained within the “Exercise Booklet” so that they can provide real-time demonstration of exercises to participants during video-conferencing, using the share-screen feature of Zoom. Participants do not have access to the online video library outside of the consultations. Video-conferencing consultations are video-recorded via Zoom and files are stored on a secure password-protected cloud-based system.

#### Treatment fidelity

Bespoke semi-structured consultation notes (paper-based or electronic according to physiotherapist preference) will be recorded by the physiotherapist for each consultation and returned to research staff at the completion of the participant’s series of consultations. Notes will be scrutinised by research staff for physiotherapist adherence to trial protocols. Fidelity will be recorded as the number and proportion of i) strengthening exercise programs prescribed by the second consultation; ii) physical activity plans prescribed by the second consultation; iii) follow-up consultations that reviewed strengthening programs; iv) follow-up consultations that reviewed physical activity plans; v) consultations where the RPE for the strength program was between 5 and 7; vi) all consultations that included education. The mean (SD) RPE for each participant’s strengthening exercise program will be determined over their consultations, as recorded in the treatment notes.

### Outcome measures

Table 4 summarises the schedule of enrolment, interventions and outcome measures for this RCT according to SPIRIT recommendations [16].

**Table 4:**
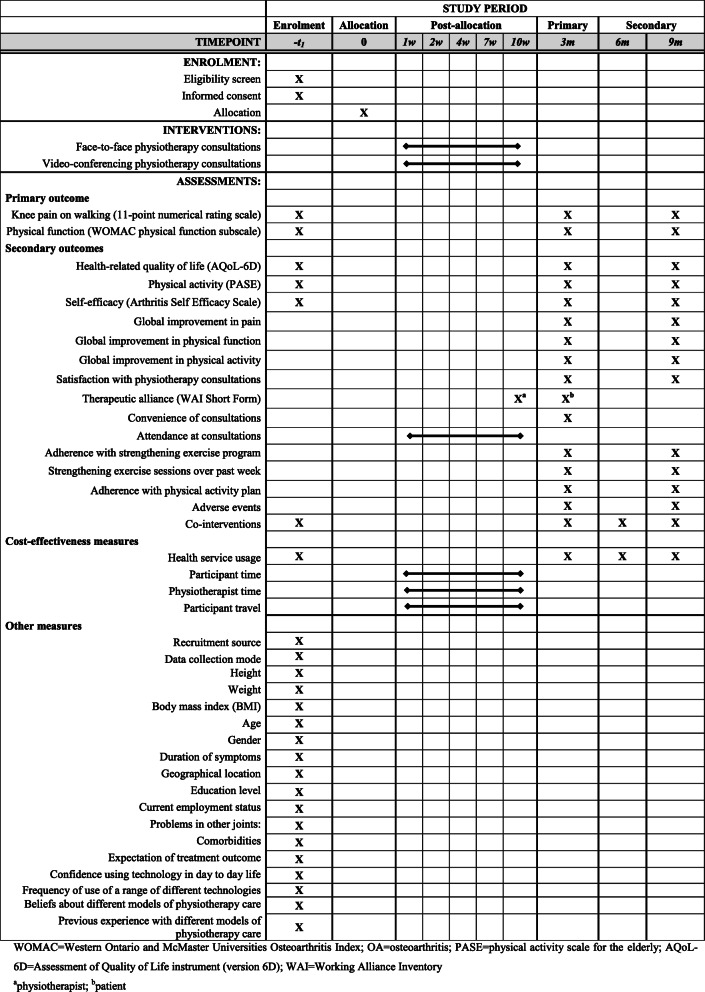
Schedule of enrolment, interventions and assessments

#### Primary outcomes

There is no single common symptom that drives all people with knee OA to seek care from a physiotherapist. However, the two predominant reasons are for relief of knee pain and/or assistance with physical dysfunction [31, 32]. Furthermore, assessing both of these symptoms as quality indicators for the primary care of OA, is advocated [33, 34]. Thus we have chosen co-primary outcomes of change in i) pain; and ii) physical function, both of which are also specifically advocated for use in clinical trials in people with knee OA [35, 36]. The primary end-point is 3 months and the secondary end-point is 9 months. Change scores at each end-point are calculated using data measured at baseline, 3 months and 9 months using the following tools:

#### Severity of knee pain during walking

Average pain on walking in the last week is measured using an 11-point NRS with terminal descriptors ‘no pain’ (score = 0) and ‘worst pain possible’ (score = 10) from baseline, 3, and 9 months. This scale has demonstrated reliability in OA [37].

#### Physical function subscale of the Western Ontario and McMaster universities osteoarthritis index (WOMAC)

The Western Ontario and McMaster Universities (WOMAC) Osteoarthritis Index (Likert version 3.1) is used to assess limitations with physical functioning [38]. The self-reported tool is a disease-specific instrument which has established validity, reliability and responsiveness in an extensive range of OA studies [39]. The subscale contains 17 questions on knee function over the past week, with Likert response options from ‘no dysfunction’ (score = 0) to ‘extreme dysfunction’ (score = 4). Total score ranges from 0 to 68, with higher scores indicating worse function.

#### Secondary outcomes

For quality of life, physical activity and self-efficacy, change scores are calculated using data measured at baseline, 3 months and 9 months. Other secondary outcomes are measured at time-points as indicated below:

#### Health-related quality of life

Health-related quality of life is evaluated using the Assessment of Quality of Life (AQoL) (version AQoL-6D) [40]. The AQoL-6D contains 20 items that assess independent living, mental health, relationships, pain, coping and senses. Total scores range from − 0.04 to 1.00, with higher scores indicating better quality of life.

#### Physical activity levels

Physical activity over the previous week is assessed using self-report tool, the Physical Activity Scale for the Elderly (PASE) [41]. Total PASE scores range from 0 to over 400, with higher scores indicating greater physical activity.

#### Self-efficacy

Using the 8-item Arthritis Self Efficacy Scale [42], participants rate how certain they are that they can do 8 tasks using response options ranging from 1 (very uncertain) to 10 (very certain). Total scores are an average of the 8 items with a range from 1 to 10; higher scores indicate higher self-efficacy.

#### Participant-perceived global change

Participants rate their overall global i) change in pain, ii) change in physical function, and iii) change in physical activity at 3- and 9-months using separate 7-point Likert scales with terminal descriptors of ‘much worse’ to ‘much better’ [43]. For each scale, participants that indicate they are ‘moderately better’ or ‘much better’ are classified as ‘improved’ and all others as ‘not improved’.

#### Satisfaction with the physiotherapy consultations

Participants rate their satisfaction with the physiotherapy consultations at 3- and 9-months using a 7-point Likert scale with terminal descriptors of ‘extremely unsatisfied’ to ‘extremely satisfied’. Participants who report being ‘moderately satisfied’ or ‘extremely satisfied’ are classified as ‘satisfied’ and all others as ‘not satisfied’.

#### Therapeutic alliance

The Working Alliance Inventory Short Form [44] is scored separately by both the participant (at 3-months) and the physiotherapist (after the 5th consultation or on the day the 5th consultation was due to be scheduled for participants who cancel/do not attend). Overall scores range from 12 to 84, with higher scores indicating a stronger therapeutic alliance.

#### Convenience

Participants rate the convenience of their physiotherapy consultations at 3 months using an 11-point NRS with terminal descriptors of ‘extremely inconvenient’ (score = 0) and ‘extremely convenient’ (score = 10).

#### Attendance at consultations

Attendance at each consultation is recorded by physiotherapists in treatment notes, and by participants in an “Appointment Log Book”. The number of consultations attended, number of consultations cancelled/rescheduled, and number of consultations that were a “failed to attend” are reported throughout the 3-month intervention period.

#### Adherence with strengthening exercise program

At the 3-month and 9-month follow-up assessments, participants rate their adherence with their prescribed strengthening exercise program (“I have been doing my exercises exactly as I was asked to by my PEAK trial physiotherapist (number of sessions, exercises and repetitions)”) using an 11-point NRS (with terminal descriptors of ‘strongly disagree’ (score = 0) and ‘strongly agree’ (score = 10)). At each time-point, participants also self-report the number of strengthening exercise sessions performed over the previous week.

#### Adherence with physical activity plan

At the 3-month and 9-month follow-up assessments, participants rate their adherence with their physical activity plan (“I followed the physical activity plan that my PEAK trial physiotherapist helped me to develop”) using an 11-point NRS (with terminal descriptors of ‘strongly disagree’ (score = 0) and ‘strongly agree’ (score = 10)).

#### Co-interventions

Participants self-report any co-intervention use (medications for knee pain and any other treatments for knee OA) at baseline, 3, 6 and 9-months as part of the custom-developed survey collecting health service usage data (see below).

#### Adverse events

Participants who experience any adverse outcomes are instructed to discuss these with their physiotherapist, who institutes appropriate advice and/or changes to the strengthening exercise program and/or physical activity plan. Any risks to participants are likely to be minor and transient. In this trial, adverse events are defined as any problem experienced in the study knee or elsewhere in the body deemed by the participant to be a result of the exercises, physical activity plans and/or advice given by the physiotherapist AND at least one of i) that caused increased pain and/or interfered with function for 2 days or more, and/or ii) resulted in the participant seeking treatment from a health professional. Adverse events are ascertained by survey questions to participants at 3 and 9 months. The number and type of events are reported.

#### Cost-effectiveness outcomes

##### Health service usage

Participants retrospectively recall their health service usage for their knee pain and/or as a result of trial participation over 3-month intervals. Participants complete a custom survey to indicate the frequency of visits to health care providers for their knee pain, use of prescription and over the counter medication, injections, hospitalisation and investigative procedures at baseline, 3, 6 and 9 months.

##### Participant time

Participants record their total time spent per consultation (including travelling to/from, waiting and consultation time) in an Appointment Log Book. Mean time for consultations is reported.

##### Physiotherapist time

Physiotherapists record their total time spent per consultation (excluding note-taking and appointment scheduling). Mean time for consultations is reported.

##### Participant travel

Distance travelled to attend consultations, along with mode of transport (including vehicle descriptions and need for another person to accompany (if applicable)), is recorded by participants in an Appointment Log Book. Mean distance travelled, mode of transport and need for another person is reported.

##### Descriptive measures

A range of participant self-reported descriptive measures are recorded at baseline including recruitment source; data collection mode; height; body mass; body mass index; age; gender; duration of symptoms; geographical residential location; education level; current employment status; problems (pain, aching, discomfort or stiffness) in other joints; comorbidities (via the Self-Administered Comorbidity Questionnaire [45]); expectation of treatment outcome (rated on a 5-point ordinal scale with anchors of “no effect at all” to “complete recovery”); confidence using technology in day to day life; frequency of use of a range of technologies; beliefs about different models of physiotherapy care delivery and previous experience with different models of physiotherapy care delivery.

### Sample size calculations

Sample size is based on detecting non-inferiority of video consultations relative to face-to-face at 3 months after randomisation. For change in NRS pain, a non-inferiority margin (NIM) of 0.95 units was chosen as this is less than the lowest of the range (1.0–2.0 units) [46, 47] reported as the minimum clinically important difference (MCID) by people with chronic pain, and less than the MCID of 1.75 units (extrapolated from a 100 mm visual analogue scale (VAS)) [48] for OA by clinician consensus. For change in WOMAC subscales, a NIM of 8 mm on VAS versions of WOMAC (score 0–100) is used in drug non-inferiority RCTs [49], as it is less than the MCIDs of 9.1–9.3 mm [50, 51]. We are using the Likert version of WOMAC (scored 0–68) for function, thus our NIM for change in function is 5.44 units (extrapolated from 8 mm). Assuming standard deviations (SD) of changes from baseline of 2.8 and 15 units for pain and function respectively and correlations of 0.3 between baseline and follow-up [11, 26], 15% loss to follow-up, 90% power, and a one-sided 2.5% significance level, we need 197 people/arm for change in pain and 172/arm for change in function, a total of 394 people.

### Statistical analysis plan

The a priori statistical analysis plan is described below. Any future amendments to this statistical plan will be documented, dated and explained in an electronic log kept by the research team, which will be made available upon request. A biostatistician will perform data analyses.

Consistent with OARSI guidelines [52], non-inferiority will be assessed using the per-protocol dataset (including only those randomized participants who attended ≥3 consultations). Between-group differences in mean change in pain and function (baseline minus follow-up) will be compared using linear regression modelling adjusted for baseline and the stratifying variable of physiotherapist. If a physiotherapist treats participants at two locations, two separate terms for that physiotherapist, corresponding to each location, will be included. Non-inferiority will be demonstrated if the lower bound of the two-sided 95% CI for between-group difference (video-conferencing minus face-to-face) is above − 0.95 for change in pain and/or − 5.44 for change in function, at 3 months. 95% CIs correspond to testing the null hypothesis of non-inferiority at a one-sided significance level of 2.5%. Due to difficulties with their interpretation, *p*-values associated with non-inferiority hypotheses are not commonly reported and will not be reported here [53]. Multiple imputation will be used to impute missing data, and an intention-to-treat analysis (including all participants in their randomised groups) will be conducted to help assess robustness of conclusions (sensitivity analysis). Given that participants with knee OA seek physiotherapy care for different reasons, we will interpret and report findings transparently and separately for each co-primary outcome. For example, if we demonstrate non-inferiority of video consultations with respect to function but not pain, we will conclude that video consultations are non-inferior to face-to-face care for improving function but are inferior for pain relief in people with knee OA. Patients seeking care, and physiotherapists delivering care, will then be fully informed about benefits, and limitations, of video consultations compared to face-to-face care with respect to the different co-primary outcomes. If non-inferiority of a co-primary outcome is demonstrated, superiority of the outcome will then be assessed using the intention-to-treat dataset and declared if the lower bound of the two-sided 95% CI for between-group difference exceeds zero.

Secondary outcomes will be assessed using data from all participants (i.e. the intention-to-treat sample) and confidence intervals will be interpreted using the superiority framework since we have not pre-defined any NIMs for these outcomes. Linear regression models will be fit to compare continuous outcomes; proportional odds models to compare improvement based on global change; and Poisson or negative binomial regression models to compare count outcomes as appropriate. Assumptions will be assessed using standard diagnostic plots and methods. Since these are all secondary analyses, *p*-values for these assessments will not be adjusted for multiple comparisons [54, 55]. In addition, the outcomes of participant time, physiotherapist time and travel time will be compared between treatment groups using the superiority framework, similarly to other secondary outcomes.

Irrespective of the outcomes of the non-inferiority analysis, we will conduct exploratory analyses to evaluate moderation of the effect of video-conferencing versus face-to-face consultations on primary outcomes by pre-specified potential moderators, i) experience with online video platforms; ii) geographical residence; iii) beliefs about physiotherapy care delivery; and iv) confidence using technology. This will be assessed by including appropriate interaction terms between the moderators and the intervention term, where the superiority framework will be applied for interpreting results. The a priori hypotheses to be tested are:

i. Participants who are less frequent users of video platforms will have less improvement in primary outcomes with video consultations (relative to face-to-face), compared to participants who are more frequent users.
ii. Participants who don’t live in major city areas will report greater ease of access, and reduced participant-level time and financial costs (secondary outcomes) with video consultations (relative to face-to-face), compared to participants who live in major cities.
iii. Participants who believe that video consultations are less effective for managing musculoskeletal problems will report less improvement in primary outcomes with video consultations (relative to face-to-face), compared to those who believe video consultations are more effective.
iv. Participants who are less confident with using technology will report less improvement in primary outcomes with video consultations (relative to face-to-face), compared to participants who are more confident.

### Economic evaluation

A health economist will oversee assessment of incremental direct and indirect costs of video consultations compared to face-to-face. Primary evaluation will be between-group difference in knee-related health care costs and quality-adjusted life years (QALYs) using generalised linear models to adjust for baseline. Non-inferiority in QALYs will be demonstrated if the lower bound of the two-sided 95% CI for between-group difference of the AQoL-6D is above − 0.08 (half a SD). If one type of consultation is superior but costs more, QALYs will be calculated using area under the curve over 9 months. The incremental cost per QALY as the ratio of difference in mean cost to difference in mean QALYs, and net benefits as the difference in QALYs times the social value of a QALY minus the difference in cost, will then be calculated. If non-inferiority of QALYs is demonstrated, then inferiority in terms of cost (and net benefits) will then be assessed if the lower bound of the two-sided 95% CI for between-group difference exceeds zero. In a secondary analysis, the cost of patient time will be included as an additional cost component.

### Patient and public involvement

No patients were involved in setting the research question. Patients with knee OA were involved in i) review of the plain language statement and consent form, particularly the text related to limited disclosure; ii) review and completion of the electronic outcome measurement survey; iii) testing the participant instructions for, and use of, the wearable activity tracker; iv) testing participant instructions for, and use of, Zoom for video-conferencing; v) review of other participant resources (Osteoarthritis Information, Exercise Booklet) and the Appointment Log Book used in data collection; vi) creation of telehealth training videos for the physiotherapist e-learning modules, and exercise videos/photographs for the website/Exercise Booklet, as patient actors; and vii) review of study logo. Patients provided feedback to the research team about information content, presentation and readability, as well as time taken to complete the outcome measurement survey. Patients with knee OA also participated in pilot “practice consultations” with the trial physiotherapists as part of the physiotherapist’s training procedures.

Representatives of the national physiotherapy professional body (Australian Physiotherapy Association) were involved in designing the trial (including selecting outcome measures) and obtaining funding. The Australian Physiotherapy Association assisted in physiotherapist recruitment by advertising for physiotherapists to participate in the trial via member electronic communications. Physiotherapists (not otherwise involved in the trial) i) reviewed the exercises contained within the “Exercise Booklet” and suggested the addition of three new balance exercises; ii) reviewed and provided feedback on the physiotherapist “Zoom Troubleshooting” resource embedded within the e-learning modules and physiotherapist trial manual; and iii) performed as actors/models in the creation of telehealth training videos for the physiotherapist e-learning modules.

The trial protocol underwent independent review by the ANZMUSC Clinical Trials Network, which included a written submission and a verbal presentation in an open forum to ANZMUSC members, review by two members from each of the Scientific Advisory Committee and the Consumer Advisory Group. The trial was endorsed by ANZMUSC on 25/07/2019, indicating its high priority and quality, importance to consumers/patients, clinicians and policy makers, and its potential to improve patient outcomes.

### Timelines

Ethics approval was obtained from the University of Melbourne Human Research Ethics Committee in May 2019. Physiotherapists were recruited and underwent training (on a rolling basis) between October 2019 and March 2020. Participant recruitment commenced in November 2019 and is anticipated to be completed in August 2021 but may be extended depending on the impact of the COVID-19 pandemic on recruitment rates. The trial is currently anticipated to be completed by May 2022, when all participants are currently anticipated to have completed 9-month follow-up.

## Discussion

Although our prior research [11] showed that video consultations with a physiotherapist (combined with pain coping skills training) are efficacious compared to online education, it remains unclear how effective video consultations are compared to traditional face-face physiotherapy care. Physiotherapy is viewed by the public and the wider healthcare community as a profession associated with “hands-on” and “physical” treatments. Foster and Delitto [56] have described how perceptions of physiotherapy professional culture form, and how patient preferences and expectations drive clinician practice and beliefs. Patients often expect to receive manual therapy treatments when consulting a physiotherapist, and physiotherapists themselves have traditionally been trained in a biomedical approach emphasising “hands-on” assessment and treatment techniques, despite the limited evidence for these approaches in many chronic musculoskeletal conditions, including knee OA. Robust high-quality non-inferiority RCTs are required to provide evidence that video consultations are non-inferior to face-to-face care in order to drive changes in service delivery models and funding policy.

Our trial is robust and will provide high-quality evidence about the effectiveness of video-conferencing compared to face-to-face physiotherapy care. Most pragmatic RCTs are superiority trials, which assess if a new approach is more effective than another standard intervention. In contrast, non-inferiority trials assess whether the effects of the new approach are within a predefined clinically acceptable margin of the effects of the standard approach [57]. Non-inferiority RCTs typically require larger sample sizes than superiority trials because the NIM is typically smaller than the treatment effect that a similar superiority trial comparing the standard treatment to a comparison/placebo would be powered to detect. The study power of non-inferiority trials is usually chosen to be high (usually 90%) to minimise the risk that a non-inferior treatment is missed due to chance [57]. Within our RCT, we plan to conduct a linked discrete choice experiment exploring patient preferences for receiving physiotherapy care, as well as qualitative evaluations of i) physiotherapist experiences with training for the trial; and ii) physiotherapist and patient experiences with, and attitudes towards, video consultations. These linked studies will be reported separately from the main trial findings.

A systematic review and meta-analysis evaluated the effectiveness of treatment delivered via telerehabilitation for musculoskeletal conditions [58]. Of the 14 trials included, only three in people with OA were identified (one of these with a sample of mixed arthritic diagnoses) and none investigated video consultations. All three were superiority trials that utilized the telephone for consultations, and only one evaluated physiotherapy. Although a large equivalence trial showed that telephone-delivered physiotherapy care was equally clinically effective as usual care in the UK [59], a diverse sample of participants with mixed musculoskeletal conditions was recruited and video consultations were not evaluated. The paucity of literature evaluating the effectiveness of video consultations for management of knee OA is a barrier to its implementation as an alternative method of delivering care by physiotherapists. A significant limitation is the absence of data about its cost-effectiveness.

The Australian Telehealth Society has called for increased research to better understand the economic benefits of telehealth, particularly for patients and the broader health system, and not just funders of services [60]. In its National Telehealth Strategy, the Australian Telehealth Society has also recognized the need to investigate the impact of telehealth on consumers, arguing telehealth will not succeed unless consumers can access it and find value in it [61]. Key actions in this Strategy include determining consumer attitudes, enablers and barriers to telehealth and assessment of consumer feedback from telehealth trials. Findings from the PEAK RCT will provide valuable evidence about the clinical effectiveness and cost-effectiveness of video-conferencing by physiotherapists for people with knee OA. Results will inform the development and implementation of telehealth physiotherapy models and will have relevance to other chronic musculoskeletal diseases where education, exercise and physical activity are a cornerstone of management. Trial findings will be particularly relevant in light of the COVID-19 pandemic which has prompted many health services globally to switch from face-to-face consultations to video-conferencing in order to protect the health of both patients and staff.

## Data Availability

The datasets used and/or analysed during the current study will be made available from the corresponding author on reasonable request.

## Abbreviations

ANZMUSC: Australia & New Zealand Musculoskeletal Clinical Trial Network
AQoL: Assessment of Quality of Life
CI: Confidence Interval
CONSORT: Consolidated Standards of Reporting Trials
HREC: Human Research Ethics Committee
MCID: Minimally clinically important difference
NIM: Non-inferiority margin
NRS: Numerical rating scale
OA: Osteoarthritis
OARSI: Osteoarthritis Research Society International
PASE: Physical Activity Scale for the Elderly
PLS: Plain Language Statement
RCT: Randomised controlled trial
SD: Standard Deviation
SPIRIT: Standard Protocol Items: Recommendations for Intervention Trials
VAS: Visual analogue scale
WOMAC: Western Ontario and McMaster Universities
